# The wide-awake local anesthesia no tourniquet (WALANT) technique in thumb injuries: a systematic review

**DOI:** 10.1007/s00068-024-02579-8

**Published:** 2024-07-05

**Authors:** Maria do Rosario Saraiva, Olalla Saiz Vázquez, Juan Hilario Ortiz-Huerta, Montserrat Santamaría-Vázquez

**Affiliations:** 1 Health Sciences Department, Universidad de Burgos, Paseo Comendadores s/n, Burgos, 09001 España; 2 School of Health Sciences of Polytechnic of Leiria, Campus 2 - Morro Do Lena, Alto Do Vieiro - Apartado 4137, 2411-901 Leiria, Portugal; 3 Department of Rehabilitation, Local Health Unit - Guarda Hospital, Guarda, Portugal

**Keywords:** (DeCS terms) pain, Thumb, Local anaesthesia, Tourniquet, Wounds and injuries, General anaesthesia

## Abstract

Human hands have a complex anatomical structure. The thumb, being an integral part of the hand, has an essential function in gripping. In this sense, thumb fractures account for 4% of all hand fractures (it may occur in association with fractures of the trapezium). The majority of hand fractures should be treated non-surgically and surgeons must avoid unnecessary surgery. Historically, hand surgery has used a combination of local/regional/general anaesthesia and a tourniquet. This study aims to carry out a systematic review to determine whether the WALANT technique is an advantageous alternative to conventional anaesthesia for surgical procedures on thumb injuries, in terms of patient function and pain. Method: We conducted a search in the following databases: Pubmed/Medline, EBSCOhost, Web of Science, Scopus, ScienceDirect and Google Scholar, using the equation "WALANT" OR "Wide Awake Local Anesthesia No Tourniquet" AND "thumb pathology". Results: In five of the 584 articles included, two studied trapeziometacarpal osteoarthritis, one De Quervain's disease and the remaining two flexor injuries. WALANT showed good results in active movements, but with similar levels of pain between anaesthetics. Patients were more anxious during general anaesthesia, plus the fact that they were fasting and suspending medication. Conclusion: WALANT is a convenient and favourable option in several studies. It has been demonstrated the benefits in terms of return to function and pain.

## Introduction

Human hands have a complex anatomical structure and are the main limbs for carrying out activities of daily living [1]. The physical and tactile capabilities of the hands allow to perform a variety of tasks and movements [1]. The thumb, being an integral part of the hand, has an essential function in gripping [2]. It is responsible for approximately 60 per cent of the gripping function, which can be attributed to its position [3]. Its uniqueness and versatility are mainly due to its biomechanics, allowing for opposability, which consists of opposition and gripping, however, this position also exposes the thumb to unique injuries [4, 5].

The most common traumatic injuries include fractures, dislocations, injuries to the collateral ligaments of the metacarpophalangeal joint (MCF) and other soft tissue injuries can often be found [2], such as De Quervain's syndrome. It is one of the most common causes of pain and disability [6]. Osteoarthritis of the base of the thumb or Trapeziometacarpal (TMC) osteoarthritis, better known as rhizarthrosis, also has a very common presentation [7], with a prevalence of between 7 and 35% in the general population [8] and is the second most affected joint in the hand [9].

Hand fractures account for 19% of all fractures, predominantly in the working population [10] and thumb fractures account for 4% of all hand fractures (it may occur in association with fractures of the trapezium). Young men and elderly women are most susceptible. The AO/ASIF Comprehensive Classification of Fractures is a logical system to describe and compare hand bone fractures. It is classified the fracture on the ray of the hand involved, the bone broken and the type of fracture and correlated patterns to the age of the patients [11]. A variety of fractures can occur at the thumb metacarpal base: Displaced intra-articular fractures (in addition to more comminuted variants); Bennett fractures account for 30% of all thumb metacarpal fractures; four times more frequently is the Rolando fracture; extra-articular fractures are also common, typically occurring at the metaphyseal-diaphyseal junction [12].

The majority of hand fractures should be treated non-surgically and surgeons must avoid unnecessary surgery [6]. In this line is also crutial to avoid unnecessarily complex procedures; if surgical intervention is chosen, it should be the simplest and the surgeon should make a careful and precise decision about the method [13]. Furthermore, this concept should be applied to the remaining thumb injuries.

Historically, hand surgery has used a combination of local/regional/general anaesthesia and a tourniquet [14]. Although the tourniquet has been accepted as an essential instrument in hand surgery, it is not without its dangers. Most complications are directly related to the duration of ischaemia and the pressure applied by the tourniquet; nerves are vulnerable to pressure and muscles to ischaemia [15]. More than 10 years ago, Canadian hand surgeon Donald Lalonde popularised local anaesthesia without a tourniquet, better known as Wide Awake Local Anaesthesia No Tourniquet (WALANT) [14]. This has been increasingly used by hand surgeons. In this technique, the surgeon performs anaesthesia on the patient using an anaesthetic mixture containing lidocaine, epinephrine and sodium bicarbonate (NaHCO3) [16], allowing intraoperative mobilisation by the patient of the operated region and avoiding functional impotence of the operated limb in the immediate and postoperative periods, without altering body image [17].

Since the choice of anaesthesia can optimise the hand rehabilitation process and prevent injuries related to surgery, and since the thumb is the finger responsible for the stability, strength and function of the hand, it is important to determine whether this technique brings benefits in terms of pain and function. Therefore, this study aims to carry out a systematic review to determine whether the WALANT technique is an advantageous alternative to conventional anaesthesia for surgical procedures on thumb injuries, in terms of patient function and pain.

## Method

This review is registered in PROSPERO: "International prospective register of systematic reviews", with registration number CRD42023429474, and was designed and prepared in accordance with "Preferred Reporting Items for Systematic Reviews and Meta-Analyses (PRISMA)” [18].

The PICO strategy used to describe all the components related to the problem identified and to structure the research question was as follows [19]:

- P (Population): Adult patients undergoing surgical procedures on various thumb injuries.
- I (Intervention): Patients undergoing surgical procedures on the thumb using the "Wide Awake Local Anaesthesia No Tourniquet (WALANT)" technique.
- C (Control): WALANT will be compared to conventional anaesthesia, which can be general/local/regional anaesthesia with tourniquet.
- O (Outcome): Function and pain.

### Search strategy

A database search was carried out between 1 April 2023 and 1 May 2023, according to a search strategy (Appendix IV) from Pubmed/Medline, EBSCOhost, Web of Science, Scopus, ScienceDirect and Google Scholar. This search had no restrictions on publication dates and the search equation was: "WALANT"OR "Wide Awake Local Anaesthesia No Tourniquet" AND "thumb pathology", since this term is broad and includes all injuries involving tendons, ligaments, muscles, bones and joints of the thumb, it was replaced by: "thumb surgery", "rhizarthrosis", "trapeziometacarpal osteoarthritis", "trapeziometacarpal joint, "carpometacarpal osteoarthritis", "carpometacarpal joint", "tendon injuries", "Thumb metacarpophalangeal", "De Quervain", "Quervain's tenosynovitis", "ligaments/injuries", "thumb ligament", "ligaments surgery", "thumb finger", "thumb injuries", "thumb fracture", "ulnar collateral ligament", "radial collateral ligament".

**Fig. 1:**
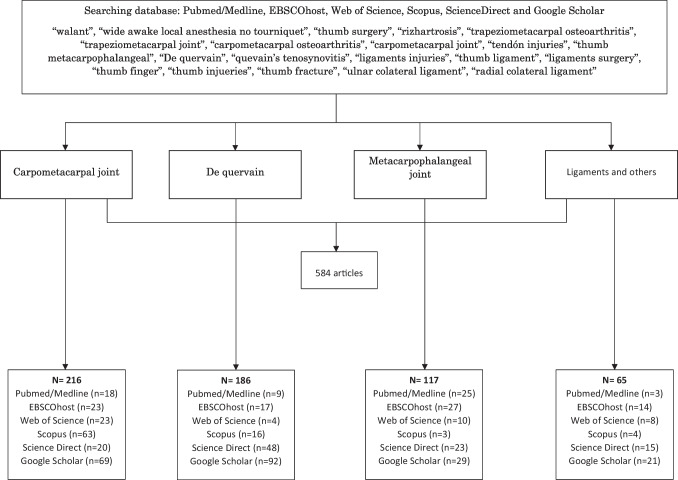
Equation for the search, firstly for WALANT and injuries to the carpometacarpal joint (CMC), then WALANT and De Quervain's syndrome, followed by a search for WALANT and injuries to the metacarpophalangeal joint (MCF) and finally WALANT and injuries to ligaments/others

The search was applied in 4 phases [18], firstly for WALANT and injuries to the carpometacarpal joint (CMC), then WALANT and De Quervain's syndrome, followed by a search for WALANT and injuries to the metacarpophalangeal joint (MCF) and finally WALANT and injuries to ligaments/others, as described in the equation shown in Fig. 1.

### Inclusion and exclusion criteria

This research had no restrictions on publication dates or journals and the inclusion criteria were: (a) studies in humans, (b) aged 18 or over, (c) undergoing surgery after thumb injury using the WALANT, (d) randomised controlled trials (RCT), experimental studies without randomisation and cohort studies, (e) with a score of 7 or more on the Critical Appraisal Skills Programme (CASP) 17 scale, (f) studies in English, French, Spanish or Portuguese. While the exclusion criteria were: (a) studies with more than one surgical intervention and/or other pathologies and comorbidities, (b) studies that did not separate the results from other clinical conditions and (c) studies using only general or local anaesthesia with tourniquet.

### Data extraction

One of the reviewers identified the articles, taking into account the title, keywords and abstracts. Duplicate articles and those that did not fulfil the inclusion criteria were excluded. Those that raised doubts as to whether or not they met the inclusion criteria were flagged for a full reading.

The articles considered to be included were extracted in full for study eligibility, independently by two reviewers. Discrepancies were resolved with reviewer 3 when necessary.

The data collected were: (1) author, year of publication and country, (2) type of injury or surgery, (3) sample inclusion criteria, (4) sample size, (5) age range of patients, (6) assessment instruments.

The main results were extracted by analysing the pain and functional assessment variables, using the NRS (Numerical Rating Scale) [20], VAS (Visual Analogue Scale) [21] Quick-DASH (Quick Disabilities of The Arm Shoulder and Hand) [22], Moineau grip and Kapandji, TAM (Total Active Motion) [23], Strickland Score and goniometry instruments [24]. The variables of satisfaction, anxiety and operating theatre times were also taken into account and measured using the EVAN-LR (Evaluation of Locoregional Anaesthetia Care) [25] scale after discharge, the HAM-A (Hamilton anxiety rating scale) [26] and the 5-point Likert Scale [27].

## Results

**Fig. 2:**
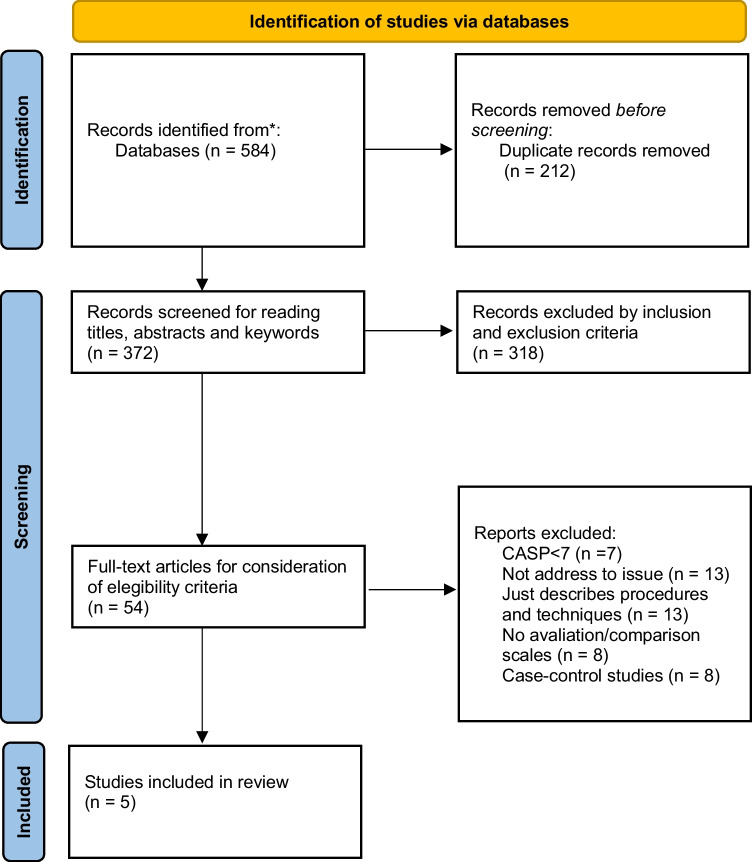
Complete screening process illustrated in the PRISMA flowchart

Of 584 articles identified, only five met the inclusion criteria and were selected for analysis after full reading. The complete screening process is illustrated in the PRISMA flowchart (Fig. 2).

Of the 54 articles, 12 were examined according to the inclusion criteria and submitted to the "Critical Appraisal Skills Program CASP: Randomised Controlled Trial Checklist" tool,17 to check the accuracy of the data, independently by two reviewers (R1 and R2). Discrepancies were resolved with a third reviewer (R3). Table 1 shows the results of the CASP, which shows that the articles are all randomised trials (RCTs). In addition to the CASP tool, the articles were classified according to the "Oxford Centre for Evidence-Based Medicine Levels of Evidence" (OCEBM) score.18 The OCEBM working group drew up "The Oxford Levels of Evidence 2011",19 in which the quality of the evidence is classified on a scale of 1 to 5, with level 1 representing the publications with the highest level of scientific evidence and 5, those with the lowest level.

**Table 1:** CASP checklist

Studies^1^ N = 5	Reviewers^2^		CASP – Checklist [25]												Score OCEBM
		Design	*Q1*	Q2	Q3	Q4	Q5	Q6	Q7	Q8	Q9	Q10	Q11	Total	
Lara Moscato et al. [9]	R1	*Single-center, comparative, retrospective, observational*	1	1	1	CT	0	1	1	1	1	1	1	**9**	**2**
	R2		1	1	1	CT	1	1	1	1	1	0	1	**9**	
Meunier et al. [26]	R2	*Prospective observational non-randomized*	1	1	1	CT	1	1	1	1	1	0	1	**9**	**3**
	R2		1	0	1	1	1	1	0	0	1	1	1	**8**	
Lee, Kim et Choy [27]	R1	*Randomized prospective cohort single- center study*	1	1	1	0	1	1	1	1	1	1	1	**10**	**2**
	R2		1	1	0	1	1	1	1	1	1	1	1	**10**	
Kadhum et al. [28]	R1	*Retrospective comparative*	1	CT	1	CT	1	1	0	1	0	1	1	**7**	**3**
	R2		1	0	1	0	1	1	0	1	0	1	1	**7**	
Kiran et al. [29]	R1	*Prospective, comparative*	1	1	1	CT	1	1	1	0	0	1	1	**8**	**3**
	R2		1	1	1	0	1	1	0	1	0	1	1	**8**	

1 N = number of studies

2 R1: reviewer 1; R2: reviewer 2

3 Q: question; 0 = NO; 1 = Yes; CT = Can't Tell

To measure agreement between the reviewers of the articles analysed, the intraclass correlation coefficient (ICC) was calculated and a value of 1.00 was obtained, which means that there is perfect agreement.

Finally, the five articles were analysed and it was found that two articles dealt with flexor injuries, two with osteoarthritis of the MCF and one with De Quervain's syndrome. No articles were included on fractures, dislocations or injuries to the collateral ligaments of the MCF of the thumb. Table 2 shows the data from the articles according to type of injury/surgery, inclusion criteria, sample, age range and assessment instruments. The main results, which deal with pain and functional assessment, are shown in Table 3 and the results complementing the study, such as satisfaction, anxiety and operating theatre times, are described in Table 4.

**Table 2:** Data from the articles according to type of injury/surgery, inclusion criteria, sample, age range and assessment instruments

Study Year Country	Type of injury/surgery^1^	Inclusion criteria	Sample (N)^3^	Mean Age^4^	Assessment tool^5^
Lara Moscato et al. [9] 2021 France	Osteoarthritis of TMC	Patients undergoing TMC arthroplasty between 1st January and 31st December 2019	N = 30 (W n = 15 e LA n = 15);	63.37	Quick-DASH. Moineau grip and Kapandji, NRS and VAS Scale from 0 (not at all satisfied) to 10 (perfectly satisfied)
Meunier et al. [26] 2022 France	Trigger finger Removal of osteosynthesis material. Arthroplasty TMC. Tenotomy Dupuytren's disease. Synovial cyst. Arthroscopy of the wrist	> 18 years old with ASA score 1–3, patients scheduled for ambulatory surgery of the elbow, wrist, hand, finger	N = 96 (W n = 48; LA n = 48) TPM: W n = 9; LA n = 6 QD: W n = 3; LA n = 10	60	EVAN-LR after discharge NRS Side effects after 7 days
Lee et al. [27] 2022 Korea	Trigger finger, CTS e De Quervain's disease	Patients diagnosed with trigger finger, CTS and De Quervain's disease	N = 169 (W n = 56, CTB n = 58, CA n = 55); DQ n = 43	59	VAS, Quick-DASH, HAM-A, 5*-point Likert Scale*
Kadhum et al. [28] 2021 United Kingdom	Flexor tendons of the hand, including the FPL	Patients with lesions of the flexor tendons of the hand included the FPL	N = 151 (W n = 53, LA n = 57, GA n = 4) W n = 4, LA n = 6 e GA n = 4	35	TAM and number of tenorrhaphy
Kiran et al. [29] 2021 Pakistan	Tendons Flexors of the hand, including the thumb flexors	Patients aged = > 15 years, with hand lesions and need for tendon suture	N = 130 (W n = 65 e GA n = 65)	29.45 W = 28,39 AG = 28,51	Strickland score Goniometer TAM second ASSH

^1^*CMC* carpometacarpal, *CMD* trapeziometacarpal, *CTS* carpal tunnel síndrome, *FPL* flexor pollicis longus

^2^*ASA* A*merican Society of Anesthesiologists*, *CMD* trapeziometacarpal, *CTS* carpal tunnel syndrome, *FPL* flexor pollicis longus

^3^*W* WALANT, *LA* local anesthesia, *GA* general anesthesia, *QD* quervain's disease, *CTB* combination of tourniquet and lidocaine solution, *CA* conventional anesthesia

^5^*EBM* evidence-based medicine, *CC* continuous certification, *ABPS* American board of plastic surgery, *EVAN-LR* evaluation du vécu de l'anesthésie locorégionale, *NRS* numerical rating scale, *Quick-DASH* quick disabilities of the arm, shoulder and hand, *VAS* visual analogue scale, *HAM-A* Hamilton anxiety rating scale, *TAM* total active motion, *ASSH* American society for surgery of the hand

**Table 3:** Main results, which deal with pain and functional assessment

Study	Pain	Functional results
Lara Moscato et al. [9]	No significant differences: in the administration of the anesthetic (*p* = 0.3); in intraoperative pain (*p* = 0.1); pain at rest (*p* = 0.4); active pain (*p* = 0.3)	QuickDASH: Significantly better in W (*p* = 0.01) Strength: No significant difference (*p* = 0.2) Kapandji score: No significant difference (*p* = 0.2) Moineau score: Significantly better in W in spherical grip (*p* = 0.01)
Meunier et al. [26]	No significant differences: Preoperative (*p* = 0.31); recovery unit (*p* = 1) and post-surgical unit (*p* = 0.44)	N/A
Lee et al. [27]	Significantly pain in CA (*p* < 0.001) Tourniquet pain in both groups was significantly lower compared to injection (*p* < 0.001) Significantly lower postoperative pain in W, (*p* < 0.001)	No significant difference in pre/postoperative function (Quick-DASH)
Kadhum et al. [28]	N/A	No significant differences in functional results according to hand therapists (*p* = 0.497) and surgeon (*p* = 0,199)
Kiran e al. [29]	N/A	Significantly better in W in the Strickland Score and TAM (*p* < 0.05) No significant differences for ROM at the sixth week (*p* = 0,101)

*N/A* not applicable, *CA* conventional anesthesia, *W* WALANT, *TAM* total active motion, *ROM* range of motion, *LA* local anesthesia

**Table 4:** Results complementing the study, such as satisfaction, anxiety and operating theatre times

Study	Satisfaction	Anxiety	Operating theatre times
Lara Moscato et al. [9]	No significant differences (*p* = 0,5)	No significant differences (*p* = 0,5)	N/A
Meunier et al. [26]	No significant differences (*p* = 0,68)	Significantly better in W (*p* = 0,03)	With significant differences, shorter time in the operating room in W (*p* = 0,01)
Lee et al. [27]	Significantly better in W and CTB than in CA (*p* < 0.001) Between CTB and W no significant differences (*p* = 0,265)	Significantly worse in AC than in CTB and W (*p* < 0,001)	No significant difference in the duration of surgery (*p* = 0.452) Significantly higher in W in preparation for surgery (*p* < 0,001)
Kadhum et al. [28]	N/A	N/A	N/A
Kiran e al. [29]	N/A	N/A	N/A

*N/A* not applicable, *CA* conventional anesthesia, *CTB* combination of tourniquet and lidocaine solution, *W* WALANT

Three articles analysed pain at different times, when the anaesthetic was administered, intraoperatively or in the recovery unit, and postoperatively. Two of the articles examined similar results in the two procedures, with no significant difference between the anaesthetics [9, 28]. Meunier et al. [28], reported that tramadol or opioids were administered postoperatively to 30 patients, 12 in the WALANT group and 18 in LA. Of the nine patients who underwent CMT surgery, only four had to take pain medication in WALANT. The third article found that pain was less intraoperatively and postoperatively with WALANT, although the pain from the injection of this method was greater than the pain from the tourniquet [29].

Regarding functional results, several important moments were considered, the first during surgery, the second in the post-operative period and finally the return to function. Lara Moscato et al. [9] compared the functional results of CMT arthroplasty under WALANT versus AL and concluded that WALANT had advantages in function (Quick-DASH), Moineau Score (ball grip) and time to return to leisure activities (AL 50.4 days and WALANT 47.4 days; *p* = 0.2). The time taken to return to activities of daily living was similar (AL 24.2 days and 19 days; *p* = 0.1). In terms of flexor tendon tenorrhaphy, Kadhum et al. [30] investigated the results of WALANT versus local anaesthesia (LA) and general anaesthesia (GA) and concluded that there were no significant differences in tendon ruptures, but revealed that there were fewer cases of flexor tendon adhesions in WALANT and subsequently no need for tenolysis. While Kiran et al. [31] evaluated the functional results in relation to range of motion in WALANT versus conventional anaesthesia (CA) and the average TAM for the thumb was 68.33 at week 6, increasing significantly to 73.33 at week 12 and concluded that the functional results in active range of motion are better in WALANT than in CA.

In their complementary results, Meunier et al. [28] report that patients treated with WALANT are discharged more quickly because they spend less time in the operating theatre.

## Discussion

Performing surgical procedures on the hand with WALANT has become increasingly popular, as it allows for safe surgery without the need for a tourniquet and sedation of the patient through the combination of lidocaine and epinephrine [32], and this review aimed to determine whether the WALANT technique would be an advantageous alternative for surgical procedures on thumb injuries, compared to conventional anaesthesia, in terms of pain parameters and patient function.

In general, WALANT showed pain levels similar to those of LA, while GA showed greater pain. In terms of function and satisfaction, the differences were not statistically significant, but patient anxiety was lower with WALANT.

Of the three articles that analysed pain, there was no uniformity in the results, which may be a consequence of the type of injury and surgical protocol, while one of the studies analysed De Quervain's syndrome, the others dealt with rhizarthrosis, and surgery for osteoarthritis of the CMT is a more complex procedure, which can involve deep tissue dissection, bone removal and the placement or not of a component and consequently be more painful regardless of anaesthesia [33]. Despite these figures, some studies report that WALANT is an advantage because it allows the patient to actively move and perform the thumb dyad during a MCT suspensionoplasty, allowing adequate tension to be confirmed [34]. With regard to pain at the time of injection being more painful than the tourniquet, this may be due to the insertion of the needle, which causes sudden, sharp pain, regardless of the solution injected [29].

As with pain, the functional results of this review do not provide sufficient data to demonstrate the advantage of WALANT, some variables (joint ranges and muscle strength) are directly related to the clinical severity of the patient, the selection of the surgical technique and the different rehabilitation protocols [35]. On the other hand, the scales and tests used to assess function are different in the studies included. With regard to the Quick-DASH questionnaire, Dacombe et al. [36]. showed that DASH has an excellent reliability profile, but it addresses the upper limb as a functional unit, which makes it debatable whether it is the most suitable for thumb injuries or more specific hand injuries. Even so, it has been found that in the first phase WALANT provides an ideal opportunity for Total Active Motion in the intraoperative period [31]. With the patient awake, it is possible to assess the movement of the thumb at the time of surgery and provide the appropriate tension under the active movement of the thumb via WALANT [37]. According to the literature, WALANT in other hand injuries allows surgeons to initiate early mobilisation and the possibility of a hand therapist taking part in the surgery to teach range-of-motion exercises and discuss rehabilitation plans [32], making it a benefit for the patient's function. Just as Lalonde [38] considers it important for the patient to first practice the permitted movements in a completely pain-free environment during surgery and under the direction of a therapist, Moriya et al. [39] also states that it is better to recover as much active range of motion as possible through early active movement than to wait for an improvement with tenolysis. These arguments are in line with the results obtained in this research, in which WALANT leads to a reduction in adhesions in the flexor tendons and consequently fewer occurrences of tenolysis [30, 31].

In terms of anxiety, the results obtained with WALANT coincide with studies that have analysed other hand injuries using this technique, such as the study by Davison et al. [40] in which anxiety was also lower. These data correlate with several factors, one of which is the lack of need for preoperative tests, another is the lack of need for intraoperative monitoring and also the patient's perception of side effects; with WALANT, patients simply leave the surgery and go home [41]. On the other hand, the study by Abd Hamid et al. [42] reported no statistically significant difference between the WALANT and GA groups, in which anxiety was assessed with the Amsterdam Preoperative Anxiety and Information Scale (APAIS). The differences in results may be related to the use of different scales to assess the same variable.

In terms of satisfaction, the results of this review do not differ between WALANT and the other anaesthetics, unlike the study by Seretis et al. [43] in which they obtained a very high satisfaction rate, especially in the WALANT group, as well as the study by Ayhan et al. [44] in which 77.5% of the patients admitted that surgery with WALANT was easier than they had expected. This disagreement in results may be related to the age of the patients, as in the articles on thumb osteoarthritis with WALANT the sample had a higher average age. The literature mentions that the older population is more likely to suffer from post-traumatic stress and anxiety, which can be exacerbated in hospital environments, while anxiety can increase the perception of pain and decrease patient satisfaction [45].

With regard to operating theatre times, while on the one hand the WALANT group reported slightly longer operative times compared to LA due to preoperative preparation, on the other hand they reported that patient time in the operating theatre was shorter and that patients were discharged more quickly compared to GA [28]. The disparity in the values found is due to the waiting time of between 26 and 30 min for the administration of the WALANT injection, as opposed to the 7 min traditionally taught. The vasoconstrictor effect of epinephrine has to act against the vasodilator effect of lidocaine and the release of histamine from the trauma of the needle and the fluid injected for haemostasis during surgery [46]. Regarding patient discharge, this is quicker with WALANT because there are no effects of deep sedation as with GA (nausea, vomiting and dizziness) and hospitalisation is considerably reduced [35].

In the studies that were included, the complications of WALANT or other anaesthetics, as well as the different concentrations of epinephrine that were administered, were not taken into account, which is one of the limitations of this review. Another limitation was the choice of study design (only RCTs), although useful in terms of conceptualisation, study design can cause variability in results. Of the studies that used WALANT in their approach [15], described surgical techniques and procedures, eight did not use comparison scales and eight were case studies, as this is an emerging technique, there seems to be a lack of evidence on the subject or the studies have not yet been completed. It is essential to carry out more in-depth research, with more reliability and evidence, as well as the need to use appropriate function scales. The articles included came from different countries, with different age groups and pathologies, as well as surgical approaches, so the lack of specific target populations and poorly defined comparators (heterogeneity of results) also meant that a meta-analysis was not possible.

Each stage of this systematic review followed a protocol, giving this study a greater degree of confidence. The question was defined in terms of population, interventions, comparators, results and study design (PICOS), which meets a topical issue, becoming the strengths of this study.

In the future, it would be worth considering carrying out studies that address a functional assessment before and after surgery, relating WALANT to the rehabilitation protocol and even determining the benefits of including a therapist specialised in hand rehabilitation in the team. The implementation of this technique by the hand surgery team is considered important for successful recovery, due to the interaction between hand surgeons, therapists and patients. This could be one of the most significant changes in an operating theatre, as those who have had the opportunity to be involved in this intraoperative communication highlight this relationship as a next step in the development of hand surgery.

## Conclusion

WALANT seems to have benefits for the patient in terms of early mobilisation of structures, faster return to home and leisure activities, as well as advantages in terms of anxiety, and may be the ideal anaesthetic technique for the presence of a hand therapist at the time of surgery and throughout the rehabilitation process.

In general, this review did not find enough data to demonstrate its benefits in terms of return to function, pain and satisfaction. However, this method can increase the quality of care and the patient's well-being, both by reducing post-surgical time and by carrying out the procedure on an outpatient basis, since there is no need for anaesthetic recovery and hospitalisation, and by recovering active range of motion and reducing adhesions.

Nowadays, the intraoperative experience and patient satisfaction are increasingly important indicators of quality in the evaluation and management of the quality of healthcare establishments.

## Data Availability

No datasets were generated or analysed during the current study.
